# Efaproxiral red blood cell concentration predicts efficacy in patients with brain metastases

**DOI:** 10.1038/sj.bjc.6603169

**Published:** 2006-06-13

**Authors:** B Stea, E Shaw, T Pintér, J Hackman, M Craig, J May, R P Steffen, J H Suh

**Affiliations:** 1 Department of Radiation Oncology, The University of Arizona Health Sciences Center, 1501 North Campbell Avenue, Tucson, AZ 85724, USA; 2 Radiation Oncology Department, Wake Forest University School of Medicine, Medical Center Boulevard, Winston-Salem, NC 27157, USA; 3 Department of Oncology, Petz Alaldár Hospital of Gyor-Moson-Sopron County, Zrinyi.u.13, Gyor H-9024, Hungary; 4 Allos Therapeutics Inc., 11080 CirclePoint Road, Suite 200, Westminster, CO 80020, USA; 5 Cleveland Clinic Foundation, Department of Radiation Oncology, Brain Tumor Institute, T28, 9500 Euclid Avenue, Cleveland, OH 44195, USA

**Keywords:** efaproxiral, RSR13, whole-brain radiation therapy, brain metastases, breast cancer, radiation sensitiser

## Abstract

Efaproxiral (Efaproxyn™, RSR13), a synthetic allosteric modifier of haemoglobin (Hb), decreases Hb-oxygen (O_2_) binding affinity and enhances oxygenation of hypoxic tumours during radiation therapy. This analysis evaluated the Phase 3, Radiation Enhancing Allosteric Compound for Hypoxic Brain Metastases; RT-009 (REACH) study efficacy results in relation to efaproxiral exposure (efaproxiral red blood cell concentration (E-RBC) and number of doses). Recursive partitioning analysis Class I or II patients with brain metastases from solid tumours received standard whole-brain radiation therapy (3 Gy/fraction × 10 days), plus supplemental O_2_ (4 l/min), either with efaproxiral (75 or 100 mg/kg daily) or without (control). Efaproxiral red blood cell concentrations were linearly extrapolated to all efaproxiral doses received. Three patient populations were analysed: (1) all eligible, (2) non-small-cell lung cancer (NSCLC) as primary cancer, and (3) breast cancer primary. Efficacy endpoints were survival and response rate. Brain metastases patients achieving sufficient E-RBC (⩾483 *μ*g/ml) and receiving at least seven of 10 efaproxiral doses were most likely to experience survival and response benefits. Patients with breast cancer primary tumours generally achieved the target efaproxiral exposure and therefore gained greater benefit from efaproxiral treatment than NSCLC patients. This analysis defined the efaproxiral concentration-dependence in survival and response rate improvement, and provided a clearer understanding of efaproxiral dosing requirements.

Brain metastasis is the most common neurologic complication of cancer, occurring in up to 170 000 individuals annually in the US (Posner, 1992; Arbit *et al*, 1995). Whole-brain radiation therapy (WBRT) is a standard treatment for brain metastases (Zimm *et al*, 1981; Egawa *et al*, 1986); however, patients may not achieve maximum benefit from WBRT owing to tumour hypoxia, which decreases radiation sensitivity of solid tumours (Rampling *et al*, 1994; De Santis *et al*, 1998).

Efaproxiral (Efaproxyn™, RSR13) is a synthetic allosteric modifier of haemoglobin (Hb), which binds noncovalently in the central water cavity of the Hb tetramer (Wireko *et al*, 1991; Safo *et al*, 2001), thereby reducing the Hb-oxygen (O_2_) binding affinity to facilitate the release of O_2_ from Hb to the tissues (Randad *et al*, 1991; Khandelwal *et al*, 1993; Kunert *et al*, 1996; Watson *et al*, 1997; Steffen, 1998; Eichelbronner *et al*, 1999). This pharmacodynamic (PD) effect is measured either as a decrease in standard cutaneous pulse oximetry (SpO_2_) or as an increase in the partial pressure of O_2_ (pO_2_) to produce 50% saturation of Hb (p50). Nonclinical pharmacology (Khandelwal *et al*, 1996; Teicher *et al*, 1996; Rockwell and Kelley, 1998; Amorino *et al*, 2001) studies have demonstrated that efaproxiral can enhance the oxygenation of hypoxic tumours and function as a radiation sensitiser, increasing the effectiveness of RT.

The relationship of the E-RBC and the PD effect is well characterised (Kavanagh *et al*, 2001; Suh, 2004). The target E-RBC and resulting PD effect of efaproxiral is described best by the peak efaproxiral concentration in RBCs at the completion of the efaproxiral infusion (end-infusion); this concentration increases proportionately with dose (Venitz *et al*, 1996; Kavanagh *et al*, 2001; Venitz *et al*, 2001; Wahr *et al*, 2001). The PD target for an efaproxiral therapeutic benefit is a 10 mmHg increase in p50, which is based on a p50 increase that can be achieved and ensure ⩾90% arterial O_2_ saturation (Wahr *et al*, 2001). Clinical studies have shown that an efaproxiral dose of 75 mg/kg administered over 30–60 min often increased p50 by 10 mmHg, and a dose of 100 mg/kg consistently achieved this target (Kavanagh *et al*, 2001; Venitz *et al*, 2001; Wahr *et al*, 2001; Choy *et al*, 2005). A regression analysis that included studies in which both E-RBC and PD data were obtained at efaproxiral doses of 75–100 mg/kg demonstrated that an E-RBC concentration of approximately 483 *μ*g/ml achieved a target p50 shift of 10 mmHg. Efaproxiral exposure is calculated from the patient's E-RBC in combination with the total number of efaproxiral doses that the patient received.

The goal of the current analysis was to evaluate efficacy in relation to efaproxiral exposure in the REACH study (Radiation Enhancing Allosteric Compound for Hypoxic Brain Metastases; RT-009) (Suh *et al*, 2006). This REACH study was a randomised, Phase 3, multinational, clinical trial in patients with brain metastases from various primary cancers, which tested whether the addition of efaproxiral to WBRT (plus supplemental O_2_) would improve survival compared to WBRT (plus supplemental O_2_) alone. The primary efficacy analyses were performed on all eligible patients, as well as all eligible patients with primary tumours of either non-small-cell lung cancer (NSCLC) or breast cancer. Using unadjusted log-rank analysis, the REACH efficacy results for the co-primary populations of lung or breast primary cancer suggested an improvement in median survival time (MST) in favour of the efaproxiral arm (Suh *et al*, 2006) (although not statistically significant). After adjusting for protocol-defined prognostic factors by Cox multiple regression, the results showed a statistically significant survival advantage for efaproxiral-treated patients in both primary cancer populations compared to control patients.

In the current analysis, the E-RBC concentrations and number of efaproxiral doses administered from REACH study patients were compared with respect to primary tumour type and patient body weight. Survival and response rate in the brain were evaluated in patients who received at least 7 doses of efaproxiral, comparing those patients who achieved the target E-RBC concentration (483 *μ*g/ml) with those patients who did not reach the target (i.e., those who may have been underexposed). Because the subset of patients with primary breast cancer appeared to have a significantly improved survival outcome compared to patients of other primary tumour types, exploratory analyses of this subset were conducted to evaluate survival with respect to prognostic factors and efaproxiral exposure.

## MATERIALS AND METHODS

### Study design

Informed consent was obtained from all patients. Human-experimentation guidelines of the appropriate regulatory authorities were followed in the conduct of clinical research. Patients were randomised 1 : 1 to receive standard WBRT concurrent with supplemental O_2_ and either efaproxiral (efaproxiral arm) or no efaproxiral and no placebo (control arm). Patients were stratified to one of four strata depending on their Radiation Therapy Oncology Group (RTOG) recursive partitioning analysis (RPA) classification and primary tumour: (1) RPA Class I, (2) RPA Class II NSCLC (3) RPA Class II breast cancer, (4) RPA Class II other than NSCLC or breast cancer according to a balanced block randomization technique (Zelen, 1974).

### Treatment

All patients received a standard 2-week course of WBRT (3 Gy/fraction × 10 days), plus supplemental O_2_ (4 l/min via nasal cannula) as described previously (Suh *et al*, 2006). Efaproxiral administration began on the first day of WBRT and continued every day of WBRT, for a total of 10 doses. The protocol initially specified the first efaproxiral dose as 75 or 100 mg/kg, based on the patient's SpO_2_. A protocol amendment later specified the first dose was to be based on SpO_2_, gender, and body weight. Briefly, if SpO_2_ while breathing room air at screening (at rest and during exercise) and on WBRT day 1 was ⩾93%, efaproxiral was administered as follows: (1) male ubjects with a body weight of ⩽95 kg and female subjects ⩽70 kg (low weight (LW)) were initially administered efaproxiral at a dose of 100 mg/kg, and (2) male subjects with a body weight of >95 kg and female subjects >70 kg (high weight (HW)) were administered an initial dose of efaproxiral at 75 mg/kg. Subsequent dosing modifications (up to or decreased from 100 mg/kg) were permitted based on SpO_2_ and observed adverse events temporally related to efaproxiral administration. Dose reductions to 50 mg/kg were permitted through protocol exemptions on a case-by-case basis.

### Eligibility criteria

Enrollment was open to RPA Class I or II patients with brain metastases originating from solid tumours, excluding small-cell lung cancer, germ cell tumours, and lymphomas. Patients were required to have an SpO_2_ measurement (resting and exercise) ⩾90% and no prior treatment for brain metastases (other than resection with measurable lesion(s) remaining). Additional eligibility criteria were presented previously (Suh *et al*, 2006).

### Efaproxiral concentration in red blood cells

For measurement of E-RBC, two end-infusion blood samples were drawn from patients treated with efaproxiral: first on day 1 after efaproxiral administration, and then on a day during Week 2. Blood samples were analysed by Analytical Development Corporation (Colorado Springs, CO, USA), and efaproxiral concentrations were determined using a validated high-performance liquid chromatography method (Kavanagh *et al*, 2001; Wahr *et al*, 2001). Efaproxiral concentration in plasma was also evaluated and substituted for analysis purposes, if no E-RBC was measured. The efaproxiral concentration in plasma : blood at the 75–100 mg/kg dose has been determined to be similar (Kleinberg *et al*, 1999, 2002; Venitz *et al*, 2001).

To more fully evaluate the E-RBC concentrations by dose and number of doses, drug concentrations were extrapolated in a linear fashion to all efaproxiral doses received. Extrapolated E-RBC was the estimated mean efaproxiral concentration over the entire 10 days of dosing and was based on the actual E-RBC measurement(s) at a specific dose(s). On treatment days in which there was no blood sample taken, the E-RBC was based on the measured E-RBC at the dose on sample days. The E-RBCs at each dose were then weighted by the number of administrations given at the specific dose and divided by the total number of administrations. For example, if a patient received a 75 mg/kg dose, but no blood sample was collected on that dosing day, then the 100 mg/kg E-RBC result was used to predict the 75 mg/kg E-RBC such that the estimated E-RBC_75_ would be calculated as 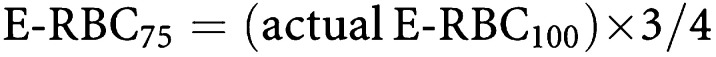


This is supported by the known linearity at these doses (Kleinberg *et al*, 1999, 2002; Venitz *et al*, 2001). If a patient then had 2 days of treatment at 75 mg/kg and eight treatments at 100 mg/kg, the extrapolated E-RBC would be calculated as 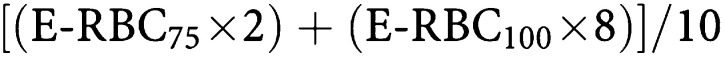


For patients who had two end-infusion E-RBC determinations at the same dose, the average of the end-infusion E-RBC determinations was used for calculating the patient's extrapolated E-RBC, and if a patient had two end-infusion E-RBC determinations at different doses, the actual E-RBC evaluations were used.

To categorise patients who had at least seven total doses of efaproxiral into high- and low-exposure groups, the extrapolated method was used to obtain an associated E-RBC for each individual dose. Patients who had seven or more doses with an associated efaproxiral concentration ⩾483 *μ*g/ml were categorised in the high-exposure (High E-RBC) group; conversely, patients who had ⩾7 total doses, but less than seven doses with an associated efaproxiral concentration ⩾483 *μ*g/ml were categorised in the low-exposure (Low E-RBC) group. Patients who had ⩾7 total doses but did not have E-RBC evaluations were placed in the Low E-RBC group by default.

### Efficacy assessments

The primary efficacy end point was overall survival, measured from the time of randomisation until death or the censoring date. Response rate, defined as best response (complete (CR) or partial response (PR)) post-WBRT, was evaluated as a secondary end point. Response evaluations and criteria have been described previously (Suh *et al*, 2006).

### Analyses and statistical considerations

The survival analysis was performed using a two-sided log-rank test (unadjusted for covariates). 31 January 2003 was stipulated as the cutoff (censoring date) for follow-up, as it allowed for the prespecified number of events, and permitted at least 6 months of follow-up for each patient. The covariate and treatment effects were also estimated using a Cox multiple regression model that included six baseline categorical covariates, in addition to treatment arm. The functional form and distribution of these covariates are listed in Table 1. The Kaplan–Meier method (Kaplan and Meier, 1958) was used to estimate survival over time, censoring patients alive as of the censoring date. Statistical significance was assessed using the Wald test statistic, and no approximation to the likelihood in the event of tied failure times was utilised. Treatment arm comparisons of response rate in the brain were made using the *χ*^2^ test. SAS version 8.2 was used for all analyses.

## RESULTS

### Patient disposition and characteristics

A total of 538 patients from 82 clinical research sites and 12 countries were randomised in the REACH study. Patient disposition for the study has been described previously (Suh *et al*, 2006). The E-RBC analysis focuses on three patient populations: (1) all eligible patients, (2) those with NSCLC as primary cancer, and (3) those with breast cancer as primary cancer.

The demographics of these three analysis populations demonstrated that the treatment and control arms were comparable with respect to age, gender, and racial distribution. Most patients (89%) in both treatment arms were Caucasian, and the majority (56%) of all patients in both treatment arms were female. The mean age of all patients was 57 years; however, the mean age of breast cancer patients was slightly younger than the other patient populations (Suh *et al*, 2006).

Table 1 presents the protocol-specified prognostic factors for survival by treatment arm and primary tumour site. In general, the treatment arms were well balanced. Treatment arm differences of greater than 5% in all patients were observed only for karnofsky performance status (KPS), where the efaproxiral arm had a greater percentage (by 6%) of patients with a KPS of 90–100.

### Treatment exposure

Of the eligible patients randomised to the efaproxiral arm, 97% (258 out of 265) received at least one dose of efaproxiral followed by WBRT. Of all eligible and treated patients in the efaproxiral arm, 54% (140 out of 258) received all 10 doses, and 84% (216 out of 258) received seven or more doses; the number of doses was similar for the NSCLC and breast cancer subsets. Dose reduction occurred in 47% (122 out of 258) of all patients; in most cases, patients received a dose reduction to 75 mg/kg, although 10% (26 out of 258) received reductions to 50 mg/kg. Nearly 46% (118 out of 258) of all patients had at least one efaproxiral dose omitted. The percentages of reduced and/or omitted doses were similar across patient populations. However, a higher number of breast primary cancer patients had reductions and/or omissions owing to adverse events, whereas NSCLC patients tended to have dose modifications, as required per protocol, due to reasons related to asymptomatic hypoxemia.

### Efaproxiral exposure by patient body weight category

A median of 1.6 end-infusion samples were evaluated for patients in each of the analysis populations. A majority (69%; 178 out of 258) of patients had two E-RBC samples evaluated. A total of 10 (4%) eligible, efaproxiral-treated patients had no blood sample evaluated (six NSCLC, two breast, and two other primary cancers).

Table 2 shows that all eligible patients who had an E-RBC sample evaluated after receiving 100 mg/kg efaproxiral (*n*=164) had a mean E-RBC of 581.1 *μ*g/ml. Patients who received 75 mg/kg efaproxiral (*n*=128) had a mean E-RBC of 461.3 *μ*g/ml. Breast cancer primary patients had a higher overall mean E-RBC compared to NSCLC patients at both doses. Patients who were administered a higher efaproxiral dose (100 mg/kg) had a proportionately higher mean E-RBC, compared to patients administered the lower (75 mg/kg) dose.

For the 75 and 100 mg/kg doses and for all three populations, the mean E-RBC increased with an increase in body weight category. Of significance, LW patients who were administered 75 mg/kg appeared to be inadequately dosed to achieve the target E-RBC (483 *μ*g/ml). The mean E-RBC of LW patients dosed at 100 mg/kg and of HW patients at either dose achieved the target concentration of 483 *μ*g/ml; HW patients dosed at 100 mg/kg had notably higher mean E-RBCs. A greater percentage of breast cancer patients were in the HW group (54%; 31 out of 57), compared to all patients (25%; 64 out of 258) and the NSCLC subset (17%; 24 out of 141). It therefore follows that a greater percentage of breast cancer patients had E-RBC concentrations ⩾483 *μ*g/ml.

### Efaproxiral exposure groups by primary tumour type

Of the 258 efaproxiral-treated patients, less than 17% (42 out of 258) received fewer than seven doses. The exposure analysis presented in this section only included patients with at least seven doses; a fewer number of doses would not have been expected to result in a treatment benefit.

Table 3 shows that for the 216 patients having at least seven doses of efaproxiral treatment, 55% (118 out of 216) were classified in the Low E-RBC group with a mean extrapolated E-RBC of 413.5 *μ*g/ml. The remaining 45% were classified as High E-RBC with a mean extrapolated E-RBC of 583.1 *μ*g/ml. Comparing the High E-RBC group and the Low E-RBC group for all patients, an 8% increase in mean efaproxiral dose (88.2 *vs* 81.9 mg/kg) was observed; however, the mean extrapolated E-RBC increase was greater than 40% (583.1 *vs* 413.5 *μ*g/ml).

Evaluating exposure groups by primary tumour type demonstrates that a majority of patients (56%; 65 out of 117) in the NSCLC subset were categorised in the Low E-RBC group; however, 52% (24 out of 46) of the breast cancer patients were included in the High E-RBC group (Table 3). This is interesting when considering that the mean number of doses and mean efaproxiral dose administered was slightly higher in the NSCLC subset for both exposure groups (Low 9.4 doses and 82.8 mg/kg; High 9.6 doses and 89.3 mg/kg) than in the breast cancer subset (Low 9.3 doses and 80.3 mg/kg; High 9.4 doses and 84.8 mg/kg).

### Efficacy outcomes by E-RBC category

**Figure 1:**
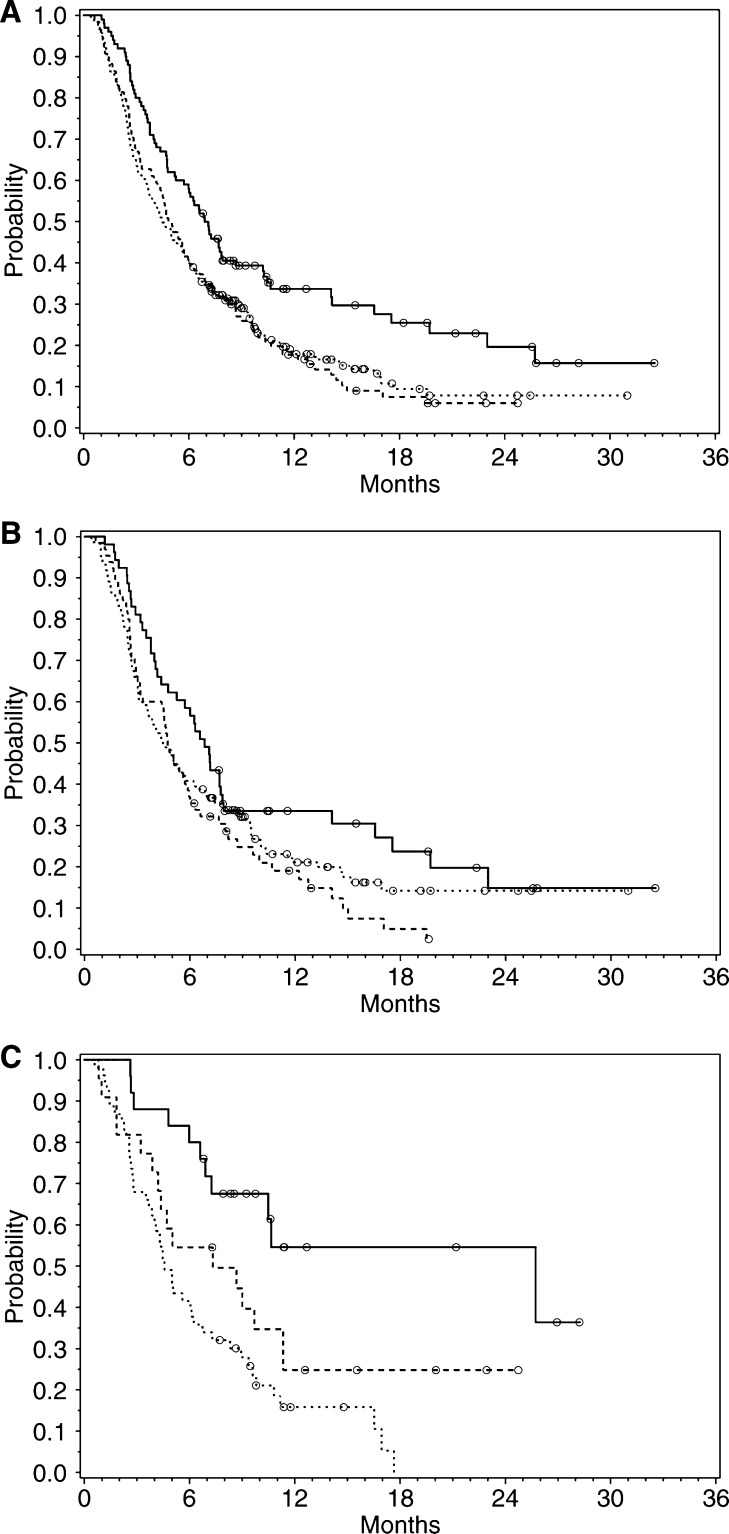
(**A**–**C**) Overall survival for all eligible patients, NSCLC patients, and breast patients by E-RBC group. The legend for the graphs below is as follows: efaproxiral High E-RBC (solid line), efaproxiral Low E-RBC (dashed line), control (dotted line), censored patients (circles). (**A**) All patients. (**B**) NSCLC patients. (**C**) Breast patients.

Table 4 and Figure 1A–C demonstrate that the High E-RBC group for each of the three populations exhibited an increased survival compared to the survival for the respective Low E-RBC groups and control arms. When analysed by log-rank test and Cox regression, the MST for the High E-RBC group demonstrated statistical significance *vs* the control arm for all patients (*P*=0.001), which was in large part driven by the breast cancer subset. In fact, the High E-RBC breast cancer subset had a 75% reduction in the risk of death based on the log-rank analysis (*P*<0.001) and a near 50% reduction based on the Cox regression analysis (*P*=0.006). Estimates of treatment effect were similar for log-rank and Cox models for the all-patient and NSCLC populations.

Exposure groups were generally well balanced for RPA class, primary disease control, age, and the presence of extracranial disease. However, the high RBC group had 14, 12, and 17% more patients with high KPS (90–100) than control for the all-patient, NSCLC, and breast populations, respectively. A solitary brain lesion was more often present in control patients than the high-exposure group for all-patients (9%) and NSCLC (14%) populations; however, 13% more of the high-exposure patients in the breast primary group had solitary lesions than control.

The breast cancer subset demonstrated a higher MST compared to all patients, as well as the NSCLC subset, for both exposure groups, but most notably for the High E-RBC group. The MSTs for the control arm were comparable across the three populations.

As shown in Table 5, a greater percentage of all patients in the High E-RBC group experienced response in the brain, compared to patients in the Low E-RBC group and to patients in the control arm. On the other hand, response rate for patients in the Low E-RBC group was comparable to control patients. A similar result was observed for patients with NSCLC. Consistent with the survival results, in the breast cancer subset, both the Low and High E-RBC groups demonstrated a response rate greater than the control arm. A better response rate was observed for breast cancer patients compared to all patients or NSCLC patients, for both the control and efaproxiral-treatment arms, which was consistent with the exposure profile.

### Breast cancer subset: efficacy outcomes by treatment arm

This analysis shows that patients with a High E-RBC had a better efficacy outcome than those with lower exposure (i.e., Low E-RBC). It was not initially clear why the patients with brain metastases originating from breast cancer outperformed the patients with NSCLC primary – the mean efaproxiral dose, mean number of administered doses, and mean extrapolated E-RBC between the respective exposure groups of these two subsets were not considerably different (Table 3).

**Figure 2:**
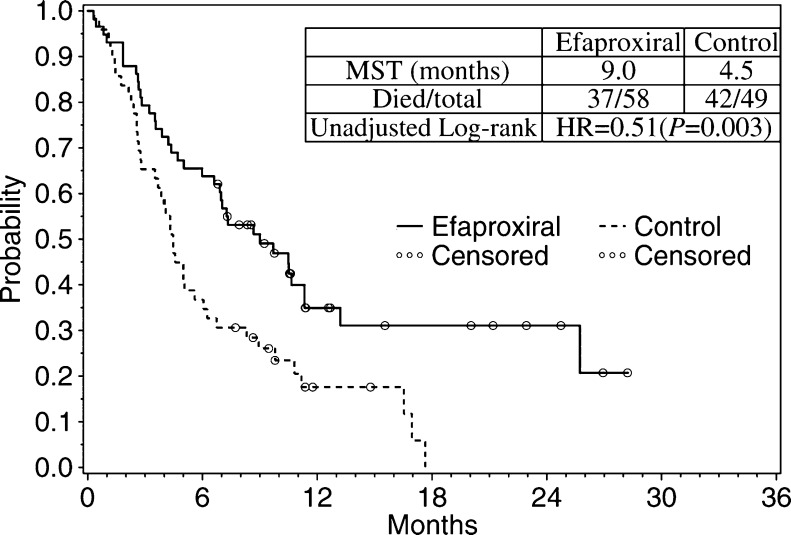
Overall survival for all eligible breast primary patients.

The exposure analysis and benefit presented above led to a separate treatment-arm analysis of the breast cancer subset. Survival analysis was performed for all eligible patients in the breast cancer subset; the Kaplan–Meier plot presenting the number of deaths and estimating survival over time is shown in Figure 2. The MST for all eligible breast cancer patients in the efaproxiral arm (*n*=58) was 9.0 months, compared to 4.5 months for the control arm (*n*=49; *P*=0.003). Furthermore, the difference in response rate for the efaproxiral arm (74%) compared to the control arm (49%) was statistically significant (*P*=0.007) for this population.

To account for confounding factors that may have affected survival in the breast cancer subset, a Cox regression analysis was performed using the six covariates that were outlined in the study protocol and presented in Table 1. As shown in Table 6, the results of the Cox regression analysis were supportive and consistent with the log-rank model demonstrating a 48% reduction in the risk of death for breast cancer patients in the efaproxiral arm. There was also a significant effect of KPS on survival, where higher baseline KPS predicted a longer survival time. When these six covariates were analysed for treatment effect by covariate subgroup and exposure group, an efaproxiral treatment effect was consistently demonstrated across subgroups (Table 7).

## DISCUSSION

The efficacy outcomes were evaluated in relation to E-RBC in the Phase 3, REACH study – one of the largest studies ever conducted in patients with brain metastases (Suh *et al*, 2006). This analysis of the E-RBC and efficacy relationship has allowed a better understanding of the therapeutic dosing requirements, which are based on gender and body weight. The information obtained from this analysis can, in part, account for the REACH efficacy results whereby there was no significant difference in overall survival between treatment arms, but patients with primary breast cancer who received both sufficient efaproxiral to achieve the target E-RBC concentration and WBRT survived significantly longer than patients who received less than the targeted efaproxiral concentration or WBRT alone (*P*=0.003).

### Efaproxiral exposure is key to positive efficacy outcomes

Considering a successful dose as one that results in an E-RBC of at least 483 *μ*g/ml and extrapolating exposure across the 10-day course of efaproxiral/WBRT, it is apparent that patients who received at least seven successful efaproxiral doses with WBRT achieved higher survival and response rates in the brain compared to patients who received less than seven successful doses, for all three patient populations analysed. Although a statistically significant difference in response rate was not observed between treatment arms in a previous analysis of all eligible patients (Suh *et al*, 2006), when analysing the patients receiving successful doses of efaproxiral *vs* control patients receiving at least seven doses of WBRT, a significant difference between treatment arms was observed (*P*=0.015). A similar observation was seen when estimating response rate by efaproxiral exposure groups (*P*=0.001). On the contrary, patients who did not receive at least seven successful doses generally had an MST and response rate similar to the control arm. However, patients with breast primary cancer were an exception in that the breast subset patients with low E-RBC also had improved survival and increased response rate compared to the respective control patients.

### Higher weight patients tended to demonstrate higher exposure

Efaproxiral E-RBC is dependent on the total dose of efaproxiral in milligrams that is administered to a patient. From the exposure analysis presented in this report, it was clear that the patients who had a higher efaproxiral exposure did not simply receive more study drug. This is supported by results showing that the all-patient population and both the NSCLC and breast cancer subsets had a disproportionate difference between the High and Low E-RBC groups in efaproxiral concentration (∼40%) compared to the difference in mean efaproxiral dose (mg/kg) administered (∼7%). Predominantly, the results show that higher efaproxiral exposure was observed for patients of higher body weight. The volume of distribution for efaproxiral is the intravascular compartment, but efaproxiral was dosed based on actual body weight, which does not correlate well with intravascular volume – the volume of distribution for efaproxiral (total blood volume) does not increase proportionately with body weight. Therefore, patients with a higher body weight received a higher total exposure to efaproxiral, defined by higher end-infusion E-RBCs, which translated to presumed greater shifts in p50 and tumour oxygenation resulting in improved RT outcomes.

### Breast cancer patients outperformed NSCLC patients

A disparity in efficacy results was observed between the patients with NSCLC *vs* breast primary cancers. The subset of patients with primary NSCLC who received the greatest efaproxiral exposure demonstrated longer survival and better response rates compared to those with lower exposure. The response rate in the brain of the high-exposure group of NSCLC primary patients approached statistical significance compared to the control arm; however, no significant difference in survival was demonstrated. Most patients in the NSCLC subset did not receive an adequate overall dose (i.e., were categorised in the Low E-RBC group), and therefore did not experience a significant efaproxiral survival benefit.

In contrast, the subset of patients with primary breast cancer demonstrated a statistically significant difference in both survival time and response rate between the high-efaproxiral-exposure group and the control arm. In addition, unlike the NSCLC subset, the low-exposure patients in the breast subset had an increased response rate and longer survival than the control group, despite not reaching significance.

A higher percentage of breast cancer patients were categorised as high body weight, and therefore achieved greater efaproxiral exposure and a greater efficacy benefit. This is evident as a majority of patients in the NSCLC subset were categorised in the Low E-RBC group, yet greater than half of the breast cancer patients were included in the High E-RBC group. This observation was not unexpected based on the previous examination of E-RBC by body weight (discussed above) in which heavier patients, in general, had higher efaproxiral concentrations, regardless of primary tumour.

### Additional study in breast cancer patient

In the REACH study, the subset of breast primary cancer patients treated with efaproxiral demonstrated a near doubling in survival (MST, control=4.5 months *vs* efaproxiral=9.0 months), which was supported by the Cox regression results and was consistent across prognostic subgroups. The survival improvement was also consistent with a significant increase in response rate in the brain (control=49% *vs* efaproxiral=74%). The information obtained from the current analysis was utilised in the development of the ENRICH study, which is an ongoing confirmatory, Phase 3 study of WBRT with or without efaproxiral, in women with brain metastases from breast cancer.

## Figures and Tables

**Table 1:** Prognostic factors for survival by treatment arm and primary site

		**Control (%)**			**Efaproxiral (%)**		
**Prognostic factor**	**Level**	**All (*N*=250)**	**NSCLC (*N*=145)**	**Breast (*N*=49)**	**All (*N*=265)**	**NSCLC (*N*=146)**	**Breast (*N*=58)**
RPA Class	I	10	10	8	11	10	14
	II	90	90	92	89	90	86
							
Primary tumour control	Yes	24	21	31	26	19	33
	No	76	79	69	74	81	67
							
Age	<65 years	73	69	80	72	69	79
	⩾65 years	27	31	20	28	31	21
							
Extra-cranial metastases	Yes	64	55	88	69	54	88
	No	36	45	12	31	46	12
							
KPS	<90	47	44	47	40	41	38
	90–100	53	56	53	59	58	62
							
Number of brain lesions	1	20	26	8	17	17	21
	2–3	32	34	18	30	36	22
	>3	47	40	73	52	46	57

NSCLC=non-small-cell lung cancer; RPA=recursive partitioning analysis; KPS=karnofsky performance status.

**Table 2:** Efaproxiral red blood cell concentration concentration by dose, weight category, and primary site for patients who received ⩾7 doses of efaproxiral

		**Low weight**			**High weight**			**All**		
		***N***	**E-RBC (*μ*g/ml)**		***N***	**E-RBC (*μ*g/ml)**		***N***	**E-RBC (*μ*g/ml)**	
**Efaproxiral dose^a^ (mg kg^−1^)**	**Primary site**		**Mean**	**s.d.**		**Mean**	**s.d.**		**Mean**	**s.d.**
75	All	92	436	101	36	527	119	128	461	113
	NSCLC	57	426	101	14	533	136	71	447	122
	Breast	12	464	71	16	519	114	28	495	100
										
100	All	138	556	138	26	716	120	164	581	147
	NSCLC	83	549	133	11	689	91	94	566	136
	Breast	21	591	124	11	754	141	32	647	150

a Because there were few patients dosed at 50 mg/kg, evaluations performed at this dose were not included.

s.d.=standard deviation; E-RBC=efaproxiral red blood cell concentration; NSCLC=non-small-cell lung cancer.

**Table 3:** Efaproxiral administered dose and extrapolated E-RBC concentration by group and primary site, for patients who received ⩾7 doses of efaproxiral

			**Administered dose**		
**Primary site**	**E-RBC group**	***N***	**Mean no. OF doses**	**Mean (mg/kg)**	**Mean extrapolated E-RBC (*μ*g/ml)**
All	Low	118	9.4	81.9	413.5
	High	98	9.6	88.2	583.1
					
NSCLC	Low	65	9.4	82.8	415.7
	High	52	9.6	89.3	579.1
					
Breast	Low	22	9.3	80.3	424.7
	High	24	9.4	84.8	597.6

NSCLC=non-small-cell lung cancer; E-RBC=efaproxiral red blood cell concentration.

**Table 4:** Median survival time by E-RBC group and primary site

	**Control**		**Efaproxiral**							
			**Low E-RBC**		**High E-RBC**					
							**Log-rank**		**Cox**	
**Site**	**D/N^a^**	**MST (months)**	**D/N^a^**	**MST (months)**	**D/N^a^**	**MST (months)**	**HR^b^**	***P* c**	**HR^b^**	***P* c**
All Patients	198/242	4.47	103/118	4.93	69/98	7.10	0.63	0.0012	0.59	<0.001
NSCLC	111/141	4.37	58/65	4.73	39/52	6.97	0.73	0.0937	0.69	0.056
Breast	41/48	4.47	16/22	7.33	10/24	25.72	0.25	0.0002	0.55	0.006

a D=number of events (deaths); *N*=number of patients.

b Hazard ratio.

c Significance test between efaproxiral, High E-RBC group, and control arm.

E-RBC=efaproxiral red blood cell concentration; MST=median survival time; NSCLC=non-small-cell lung cancer.

**Table 5:** Response rate in the brain by E-RBC group and primary site in patients who received ⩾7 doses of study treatment

	**Control**		**Efaproxiral**				
			**Low E-RBC**		**High E-RBC**		
**Primary site**	***N***	**RR^a^ (%)**	***N***	**RR^a^** **(%)**	***N***	**RR^a^** **(%)**	***P*-value (Control *vs* High)**
All patients	242	39.7	118	44.1	98	54.1	0.0153
NSCLC	141	39.7	65	43.1	52	55.8	0.0463
Breast	48	50.0	22	77.3	24	79.2	0.0174

a Response rate (RR)=complete+partial Response.

E-RBC=efaproxiral red blood cell concentration; NSCLC=non-small-cell lung cancer.

**Table 6:** Multivariate proportional hazards regression analyses for all eligible breast primary patients

**Covariate**	**Hazard Ratio**	***P*-value**
Treatment (control *vs* efaproxiral)	0.52	0.006
RPA Class (I *vs* II)	1.84	0.227
Primary tumour control (yes *vs* no)	0.88	0.658
Age	1.02	0.098
Presence of extracranial metastases (no *vs* yes)	1.09	0.829
Baseline KPS (70 *vs* 80 *vs* 90 *vs* 100)	0.73	0.029
Number of brain lesions (1 *vs* 2–3 *vs* >3)	1.05	0.796

RPA=recursive partitioning analysis; KPS=karnofsky performance status.

**Table 7:** Efaproxiral survival effect by prognostic factors and E-RBC group for all eligible breast cancer patients

		**Hazard ratio *vs* control**		
**Prognostic factor**	**Level**	**Low E-RBC**	**High E-RBC**	**All patients**
RPA class	I	Non-est^a^	0.17	0.36
	II	0.61	0.31	0.56
				
Primary tumour control	Yes	0.73	0.16	0.54
	No	0.55	0.31	0.52
				
Age (years)	<65	0.56	0.26	0.55
	>65	0.76	Non-est^a^	0.35
				
Extra-cranial metastases	Yes	0.61	0.28	0.43
	No	1.03	Non-est^a^	0.63
				
KPS	90–100	0.88	0.24	0.58
	<90	0.47	0.28	0.52
				
Number of brain lesions	1	0.39	Non-est^a^	0.36
	2	0.93	0.38	0.65
	3	0.64	0.31	0.54

a Non-estimatable due to low number of events in the efaproxiral group.

E-RBC=efaproxiral red blood cell concentration; RPA=recursive partitioning analysis; KPS=karnofsky performance status.
